# The 3' sequences required for incorporation of an engineered ssRNA into the Reovirus genome

**DOI:** 10.1186/1743-422X-3-1

**Published:** 2006-01-03

**Authors:** Michael R Roner, Joanne Roehr

**Affiliations:** 1 Department of Biology, The University of Texas at Arlington, Arlington, TX 76019, USA

## Abstract

**Background:**

Understanding how an organism replicates and assembles a multi-segmented genome with fidelity previously measured at 100% presents a model system for exploring questions involving genome assortment and RNA/protein interactions in general. The virus family *Reoviridae*, containing nine genera and more than 200 members, are unique in that they possess a segmented double-stranded (ds) RNA genome. Using reovirus as a model member of this family, we have developed the only functional reverse genetics system for a member of this family with ten or more genome segments.

Using this system, we have previously identified the flanking 5' sequences required by an engineered s2 ssRNA for efficient incorporation into the genome of reovirus. The minimum 5' sequence retains 96 nucleotides and contains a predicted sequence/structure element. Within these 96 nucleotides, we have identified three nucleotides A-U-U at positions 79–81 that are essential for the incorporation of in vitro generated ssRNAs into new reovirus progeny viral particles. The work presented here builds on these findings and presents the results of an analysis of the required 3' flanking sequences of the s2 ssRNA.

**Results:**

The minimum 3' sequence we localized retains 98 nucleotides of the wild type s2 ssRNA. These sequences do not interact with the 5' sequences and modifications of the 5' sequences does not result in a change in the sequences required at the 3' end of the engineered s2 ssRNA. Within the 3' sequence we discovered three regions that when mutated prevent the ssRNA from being replicated to dsRNA and subsequently incorporated into progeny virions. Using a series of substitutions we were able to obtain additional information about the sequences in these regions. We demonstrate that the individual nucleotides from, 98 to 84, 68 to 59, and 28 to 1, are required in addition to the total length of 98 nucleotides to direct an engineered reovirus ssRNA to be replicated to dsRNA and incorporated into a progeny virion. Extensive analysis using a number of RNA structure-predication software programs revealed three possible structures predicted to occur in all 10 reovirus ssRNAs but not predicted to contain conserved individual nucleotides that we could probe further by using individual nucleotide substitutions. The presence of a conserved structure would permit all ten ssRNAs to be identified and selected as a set, while unique nucleotides within the structure would direct the set to contain 10 unique members.

**Conclusion:**

This study completes the characterization and mapping of the 5' and 3' sequences required for an engineered reovirus s2 ssRNA to be incorporated into an infectious progeny virus and establishes a firm foundation for additional investigations into the assortment and encapsidation mechanism of all 10 ssRNAs into the dsRNA genome of reovirus. As researchers build on this work and apply this system to additional reovirus genes and additional dsRNA viruses, a complete model for genome assortment and replication for these viruses will emerge.

## Background

The name reovirus includes the acronym reo-(respiratory enteric orphan), so designated because reovirions can be isolated from the respiratory and intestinal tracts of both warm and cold-blooded animals, but have not been associated with specific clinical diseases. There are three major mammalian reovirus serotypes: serotype 1, serotype 2, and serotype 3 (ST1, ST2, ST3).

The genome of reovirus consists of ten unique segments of double-stranded RNA [1]. The segments are classified according to size. Three size classes exist: large (L) segments consist of about 3800 base pairs each, medium (M) segments consist of about 2200 base pairs each, and small (S) segments consisting of about 1100–1400 base pairs each. Each virion contains three L (L1, L2, L3), three M (M1, M2, M3), and four S (S1, S2, S3, S4) segments. Reovirions contain a transcriptase that transcribes the genome segments, by way of a conservative mechanism, into ssRNA molecules. These molecules are the (+) strand and function as mRNA. Each of the virions' RNA transcripts can code for the synthesis of at least one polypeptide. Twelve reovirus-specific polypeptides are synthesized in infected cells and are divided into three size classes: the lambda (λ) class, containing the high molecular weight polypeptides (λ1, λ2, 3), the mu (μ) class, containing the intermediate size polypeptides (μ1, μ1c, μ2, μNS), and the sigma (σ) class, containing the low molecular weight polypeptides (σ1, σ1s, σ2, σNS, 3).

The dsRNA viruses are grouped into six families: the Birnaviridae, Cystoviridae, Hypoviridae, Partitiviridae, Reoviridae and Totiviridae. Within the family Reoviridae, in addition to reovirus, extensive work on genome assortment has been done with bluetongue virus [2-4] and rotavirus [5-8]. What remains true for each of these viruses is the lack of a complete explanation for how a multi-segmented dsRNA virus is able to replicate, assort and package multiple RNA segments to yield progeny virus particles with particle to PFU ratios less than 10 and for reovirus measured by one researcher, approaching 1.0 [9].

All reovirus ssRNAs possess the tetranucleotide GCUA- at their 5' ends and the pentanucleotide -UCAUC at the 3' ends of their plus strands. This conservation of nucleotides is also present in the ssRNAs of the other members of the Reoviridae family, with all the genome segments of a specific virus possessing identical nucleotides at their 5' and 3' termini, but with these nucleotides being different from family to family.

This evolutionary conservation of the terminal nucleotides suggests a functional importance for these sequences. Work with rotavirus has identified a possible secondary structure in the rotavirus ssRNAs that involves an interaction between the 5' and 3' ends of these RNA molecules. This structure has been suggested to represent a replication/packaging structure [5]. Although such a structure may be possible for the reovirus ssRNA molecules, we have been able to demonstrate a biologically functional 5' sequence/structure [10] independent of the 3' sequence. We previously constructed a cDNA template that can be transcribed in vitro or in vivo, by T7 RNA polymerase, to yield an RNA transcript that possesses the authentic 5' and 3' terminal sequences of the reovirus s2 mRNA found in vivo. When we began to examine the 5' sequences we hypothesized that reovirus ssRNAs contain both replication signals, signals that when absent or mutated prevent the formation of a dsRNA copy of a ssRNA, and encapsidation signals, signals that when absent or mutated prevent the formation of infectious progeny virus. As our 5' data demonstrated and now our 3' data corroborates, the two signals, if they exist can not be distinguished independently from each other using our system.

The 5' sequence retains 96 nucleotides of the wild type s2 ssRNA and a predicted sequence/structure element. Within these 96 nucleotides, we identified three nucleotides A-U-U at positions 79–81 that are essential for the incorporation of in vitro generated ssRNAs into new reovirus progeny viral particles.

This work identifies the 3' downstream flanking sequence required to ensure incorporation of an engineered s2 ssRNA into the reovirus genome and, therefore, represents a major step in the process of developing a reverse genetics system for reovirus that supports the genetic engineering of any reovirus genome segment. Unlike the 5' sequence the 3' sequence contains three regions of conservation within a total requirement of 98 nucleotides. Computer secondary structure analysis has identified three possible structures that are predicted to exist in the 3' 100 nucleotides of all 10 reovirus serotype 3 ssRNAs. Using this work as a foundation we have engineered the m1 ssRNA and are currently using it to test these predictions. Publication of the sequences at the 3' termini of the reovirus s2 ssRNA will immediately allow researchers to introduce mutations into the S2 gene and protein σ2 to study the function of this protein and its role in reovirus replication and host cell interaction. Additionally, it will now be possible to use reovirus as a vaccine and gene vector by replacing the CAT gene with a gene of interest, flanking the gene with the 5' and 3' s2 sequences we have identified, and replacing the wildtype S2 genome segment in reovirus serotype 3. Researchers desiring to mutate any of the additional nine reovirus genes of serotype 3 or the genes of serotypes 1 or 2 can use these results to extend this system to these viruses. We have now completed construction of an M1-CAT reovirus using the methods and findings presented in this paper (unpublished results).

## Results and Discussion

### Construction of s2-CAT cDNA templates and transcription to yield the engineered ssRNAs

**Figure 1:**
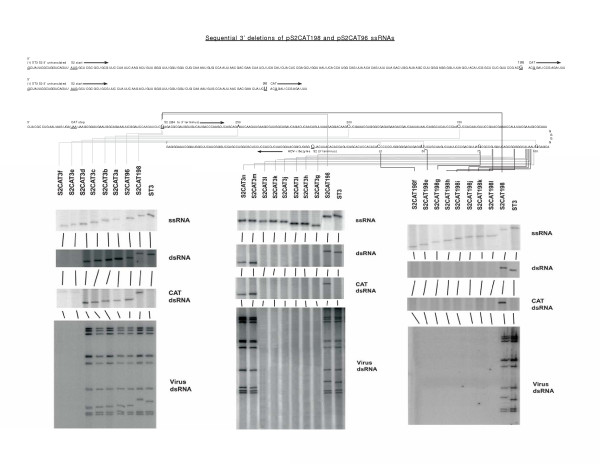
Survey of the minimum 3' terminal s2 ssRNA nucleotides required to direct a ssRNA into the reovirus genome using 50-nucleotide deletions, single-nucleotide deletions and nucleotide deletions using ssRNAs with extended 5' sequences. At the top, are the sequences of the 5' nucleotides of the ssRNAs produced using the T7 RNA polymerase promoter and cDNA template pS2CAT198 and pS2CAT96. The top two sequences include the first 18 nucleotides of the CAT coding sequence but do not show the 3' end of the ssRNA. The 3' sequence of these ssRNAs is shown in its entirety from the CAT stop codon to the authentic reovirus 3' terminus directly below the 5' ssRNA sequences. A line connects the last retained s2 nucleotide in the displayed sequence to the named cDNA plasmid, below which are displayed autoradiogram panels. Within each panel, the ssRNA, dsRNA and CAT dsRNA panels are Northerns analyzing RNA extracted from cells lipofected 12 hours earlier with 9 wildtype ssRNAs and the ssRNA obtained following transcription of the indicated cDNA template. The fourth and bottom panel of each set is an autoradiogram generated by in vivo labeling with ^32^P of the dsRNA genome segments of an isolated progeny virus containing the indicated mutated-S2 dsRNA. Progeny virus generated following lipofection was first triple-plaque purified. Deletions were initially made in blocks of 50 nucleotides. Based on the sequences required to incorporate a ssRNA into a reovirus using the ssRNAs generated from these templates, additional cDNA templates were constructed deleting ten, five and individual nucleotides until the minimal 3' sequence had been determined. Left and center panels. To test the possibility that increasing the 5' s2 sequence from 96 to 198 nucleotides might reduce the length of the 3' sequence required, a number of the 3' deleted cDNA templates were altered to include a the 198 5' sequence and retested. The ability to incorporate these ssRNAs into the genomes of reoviruses is summarized in the right panel. As described in the Materials and Methods, all ssRNAs were sequenced/analyzed to confirm the 5' and 3' ends of these RNAs.

The purpose of this investigation was to determine the 3' s2 ssRNA sequence required for incorporation into a stable recombinant reovirus utilizing our marker rescue system [10-12]. We have previously demonstrated incorporation of an engineered s2 ssRNA that retained 96 nucleotides from the s2 ssRNA 5' end and 284 nucleotides from the s2 ssRNA 3' end into the reovirus genome [10,12]. In this study, we conducted an analysis of the 284 3' terminal nucleotides of this engineered reovirus s2 ssRNA. To accomplish this, we have generated a large collection of cDNA templates based on our original templates, pS2CAT198 and pS2CAT96 [10,12]. See Figure 1.

**Figure 2:**
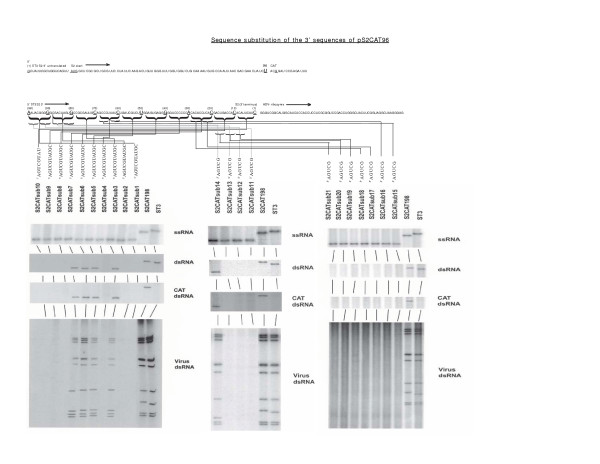
Detection of virus-generation intermediates using the ssRNAs generated from the sequence-substitution cDNA templates outlined in Table 2, using the reovirus infectious RNA system. The ssRNA (in the top panel), the dsRNA (in the next panel down) and the CAT dsRNA (the third panel down) are shown utilizing Northerns analyzing RNA extracted from cells lipofected 12 hours earlier with 9 wildtype ssRNAs and the ssRNA obtained following transcription of the indicated cDNA template. The fourth and bottom panel is an autoradiogram following SDS-PAGE generated by in vivo labeling with ^32^P of the dsRNA genome segments of an isolated progeny virus containing the indicated mutated-S2 dsRNA. Progeny virus generated following lipofection was first triple-plaque purified.

The parent plasmid contains a T7 polymerase promoter placed at the 5' end of the construct and the T7 terminator at the 3' end. The ssRNA generated from this cDNA is 1234 nucleotides long, 97 nucleotides shorter than the wt s2 RNA. It encodes a σ2-CAT fusion protein that possesses 66 σ2 AAs at its N-terminus and does not express protein σ2 function, but demonstrates CAT catalytic activity [10,12]. We use the fact that most of our recombinant viruses demonstrate CAT activity as a first screen to reduce the possibility that we have selected a serotype 2 helper virus rather than a recombinant serotype 3 virus. We also screen by SDS-PAGE analysis of the genome segments and all selected viruses are sequenced to confirm the organization of the S2 genome segment before proceeding. The remaining cDNA templates used in this study were generated using site-directed mutagenesis to delete the indicated s2 3'sequences from the pS2CAT198 or pS2CAT96 cDNA template. See Figures 1 and 2.

### 3' S2 sequences required for ssRNA incorporation

From our earlier work, we knew that 96 nucleotides from the wt s2ssRNA 5' end and 284 nucleotides from the 3' end are sufficient to enable a ssRNA to be incorporated as a dsRNA genome segment into the reovirus genome. As we have previously noted and can be seen in Figure 1, the s2CAT198 dsRNA migrates slower than the ST3 wt s2 dsRNA, although it is 97 nucleotides shorter [10,12]. This is not unexpected, as a number of reovirus dsRNA segments do not migrate in SDS-PAGE gels according to actual size.

To determine the minimal 3' s2 ssRNA sequence that retains this activity, we began by reducing the 3' sequence in steps of 50 nucleotides (ssRNAs S2CAT3a-f) from nucleotide 284. The ssRNAs generated from these cDNA templates were lipofected into cells supplying functional σ2 protein, using the reovirus marker rescue system described in the Materials and Methods section. We then examined the ssRNA, dsRNA, and CAT-dsRNA using Northerns, 12 hours following lipofection. Thirty-six hours later, samples were harvested and plaque assays performed as described. Generated viruses were isolated using plaque assays on L-ST3.S2 cell monolayers, and replaqued three times for purity. The dsRNA genome segments of individual purified viruses were labeled in vivo with ^32^P following infection of L-ST3.S2 cells and analyzed by SDS-PAGE. Autoradiograms of these cells demonstrating migration rates/patterns of individual viruses are shown in the bottom panels for each deletion. We continued with these deletions, until we reached a 3' length of 31 nucleotides. Figure 1 and Table 1.

**Table 1:** Sequential 3' deletions of pS2CAT198 and pS2CAT96 ssRNAs

**cDNA Clone**	**Length of 5' S2 sequence**	**Length of 3' S2 sequence**	**CAT Activity [pg/ml]**	**ssRNA detected**	**dsRNA detected**	**Engineered RNA incorporated into infectious virus**
pS2CAT198	198	284	64	+	+	+
pS2CAT96	96	284	64	+	+	+
pS2CAT3a	96	250	64	+	+	+
pS2CAT3b	96	200	32	+	+	+
pS2CAT3c	96	150	32	+	+	+
pS2CAT3d	96	100	64	+	+	+
pS2CAT3e	96	50	64	+	-	-
pS2CAT3f	96	31	32	+	-	-
pS2CAT3g	96	75	64	+	-	-
pS2CAT3h	96	80	64	+	-	-
pS2CAT3i	96	90	32	+	-	-
pS2CAT3j	96	95	32	+	-	-
pS2CAT3k	96	96	64	+	-	-
pS2CAT3l	96	97	32	+	-	-
pS2CAT3m	96	98	64	+	+	+
pS2CAT3n	96	99	64	+	+	+
pS2CAT198l	198	97	64	+	-	-
pS2CAT198k	198	96	64	+	-	-
pS2CAT198j	198	95	32	+	-	-
pS2CAT198i	198	90	64	+	-	-
pS2CAT198h	198	80	64	+	-	-
pS2CAT198g	198	75	32	+	-	-
pS2CAT198e	198	50	32	+	-	-
pS2CAT198f	198	31	32	+	-	-

Based on our results, the next series of deletions we made focused on the sequences between nucleotides 100 and 50. The ssRNAs, S2CAT3g-n were used to identify the minimum s2 sequence required of a ssRNA to be incorporated into a virus particle. See Figure 1. These deletions generated a ssRNA retaining 96 5' and 98 3' nucleotides from the wildtype s2ssRNA, and reduced from 1331 to 946 96 (s2-5') + 752 (CAT) + 98 (s2-3') nucleotides that are assorted, replicated to dsRNA and incorporated into a progeny virus. These results are summarized in Table 1.

### Support from additional 5' sequences

To explore the possibility that interactions could be taking place between the 5' and 3' sequences, we altered the 5' sequences of some of our 3' constructs. We examined the possibility that extending the 5' sequence from 96 to 198 nucleotides might "rescue" ssRNAs with 3' sequences less than the 98 we had just demonstrated. Summarized in Table 1 and Figure 1 using the cDNA templates pS2CAT198l-f we retested our previous 3' deletions, now with a 5' leader sequence of 198 rather than 96 nucleotides. As can be seen from our results, we were unable to "rescue" any ssRNAs with a 3' sequence of less than 98 nucleotides with a ssRNA containing 198 5' nucleotides.

### Nucleotide substitution within the required 98 nucleotides

We then generated a series of cDNA templates with 10 base substitutions of the 98 nucleotide 3' sequence we had identified. The results of these substitutions are summarized in Table 2 and shown in Figure 2. Scanning using a series of 10-base substitution sequences, we identified three regions in the 3' sequence that when substituted resulted in a loss of dsRNA synthesis and no progeny virus was produced. The first region was from nucleotides 98 to 79, clones pS2CATsub1 and pS2CATsub2. The second from nucleotides 68 to 59, clone pS2CATsub4. The third from nucleotides 28 to 1, clones pS2CATsub8, pS2CATsub9 and pS2CATsub10.

**Table 2:** Sequence substitution of the 3' sequences of pS2CAT96

**cDNA Clone**	**Length of 5' S2 sequence**	**Length of 3' S2 sequence**	**Substituted Sequence**	**CAT Activity [pg/ml]**	**ssRNA detected**	**dsRNA detected**	**Engineered RNA incorporated into infectious virus**
pS2CAT3m	96	98	none	64	+	+	+
pS2CATsub1	96	98	98-89	64	+	-	-
pS2CATsub2	96	98	88-79	64	+	-	-
pS2CATsub3	96	98	78-69	32	+	+	+
pS2CATsub4	96	98	68-59	32	+	-	-
pS2CATsub5	96	98	58-49	64	+	+	+
pS2CATsub6	96	98	48-39	64	+	+	+
pS2CATsub7	96	98	38-29	32	+	+	+
pS2CATsub8	96	98	28-19	32	+	-	-
pS2CATsub9	96	98	18-9	64	+	-	-
pS2CATsub10	96	98	8-1	32	+	-	-
pS2CATsub11	96	98	98-94	64	+	-	-
pS2CATsub12	96	98	93-89	64	+	-	-
pS2CATsub13	96	98	88-84	64	+	-	-
pS2CATsub14	96	98	83-79	64	+	+	+
pS2CATsub15	96	98	68-64	64	+	-	-
pS2CATsub16	96	98	63-59	64	+	-	-
pS2CATsub17	96	98	28-24	32	+	-	-
pS2CATsub18	96	98	23-19	64	+	-	-
pS2CATsub19	96	98	18-14	64	+	-	-
pS2CATsub20	96	98	13-9	32	+	-	-
pS2CATsub21	96	98	8-4	64	+	-	-

Using a series of 5 base substitutions we were able to obtain additional information about the sequences in these regions. Using ssRNA generated from the plasmids pS2CATsub11-21, Table 2 and Figure 2, we demonstrated that the individual nucleotides from 98 to 84, 68 to 59 and 28 to 1 are required in addition to the total length of 98 nucleotides to direct an engineered reovirus ssRNA to be replicated to dsRNA and incorporated into a progeny virion. Extensive analysis using a number of RNA structure-predication software programs, did not predict a structure that we could probe further by using individual nucleotide substitutions.

### Prediction of possible secondary structures in the 3' sequences

**Figure 3:**
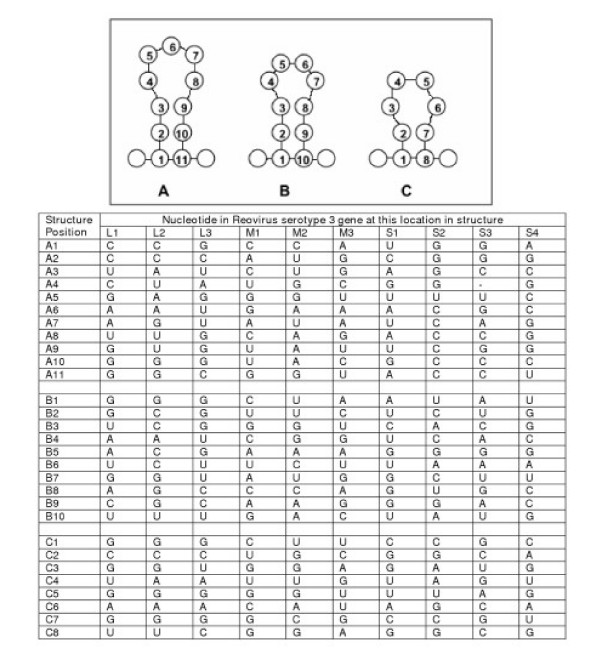
Ball and stick representation of three secondary structures predicted, using FOLDALIGN^®^, to exist in the 3' terminal 100 nucleotides of all 10 reovirus ssRNAs. Individual nucleotides present at each position in each of the 10 reovirus ssRNAs are shown in the table below the figure.

Secondary structures predicted to exist in the last 100 nucleotides of the 3' ends of all ten reovirus serotype 3 ssRNAs using FOLDALIGN^® ^[13] are shown in Figure 3.

We have continued our analysis of the reovirus s2 ssRNA to identify the sequences required to direct this RNA to be replicated to dsRNA and incorporated into the genome of reovirus. An analysis of the 5' sequences revealed a possible stem-loop structure and a requirement for 96 nucleotides retained from the wildtype s2 ssRNA [10]. These 96 nucleotides and 284 from the wt s2 at the 3' end of an engineered ssRNA are sufficient to direct incorporation of an s2 ssRNA into the reovirus serotype 3 genome. The required 3' s2 sequences have now been reduced to 98 nucleotides, a length similar to that required at the 5' end, but the overall organization of the 3' sequences appears to be quite distinct from that found in the 5' sequence.

**Figure 4:**
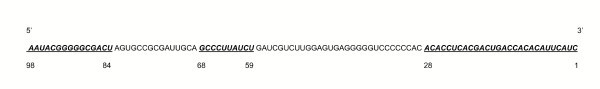
Three regions, nucleotides 98 to 84, 68 to 59, and 28 to 1, that when coupled with an additional 96 s2 5' terminal nucleotides are required to direct the incorporation of this RNA into an infectious reovirus.

We have identified three regions within a required total sequence consisting of 98 s2 3' terminal nucleotides, that when coupled with an additional 96 s2 5' terminal nucleotides are required to direct the incorporation of this RNA into an infectious reovirus. The three regions include nucleotides 98 to 84, 68 to 59, and 28 to 1. These findings are summarized in Figure 4.

When we replaced the nucleotides contained within these regions with a random sequence 5' AGUCGUAUGC or shortened versions of this sequence, we abolished the ability of the ssRNA to be incorporated as a dsRNA into the reovirus genome.

Using the RNA structure/alignment programs, RNAStructure, Vienna RNA Package, Mfold, PKnots, RNABOB and RNACAD we have been unable to obtain a predicted structure that fits our data and is also predicted to exist in the remaining 9 reovirus ssRNAs.

We have examined the possibility that the conserved 3' region may interact with the 5' sequence we have identified. To date we have been unable to identify such a structure using currently available RNA structure prediction software. Such an interaction has been proposed to function in rotavirus assembly and replication [14]. Our future examination of the sequences required in additional reovirus ssRNAs should provide the necessary data to explore such an interaction.

For reovirus to assort and assemble its ten segment genome we hypothesize that at least two types of signals exist; signals(s) that permit the RNA polymerase to bind and initiate dsRNA synthesis, and signals(s) that permit for the differentiation of each individual ssRNA as a unique member of a set of 10 RNAs. This study identifies the minimal RNA sequence at the 3'terminus required for an engineered ssRNA to be incorporated into the reovirus genome and together with our 5' data the sequences required of a ssRNA to be replicated to dsRNA and assembled into the reovirus genome. To elucidate the signals common and distinct in all 10 ssRNAs we are conducting a similar analysis of the 5' and 3' sequences of the reovirus l1 and m1 ssRNAs. With the data from these genes we should be able to identify the biological signals in the remaining seven ssRNAs and construct a model for genome assortment and replication.

Using the minimal 5' and 3' sequences we have now identified for the s2 ssRNA, it is possible to use this marker rescue system to introduce engineered mutations into the reovirus serotype 3 S2 genome segment, isolate an infectious mutant virus, and use this virus to address structure/function questions of the S2 gene and its gene product sigma 2 in vivo. In addition, at least 752 nucleotides can now be engineered into the S2 gene to produce a recombinant virus expressing an engineered protein of 250 amino acids. Reovirus can now be used to express small proteins for purification and/or vaccine development. Extension of these studies to larger genes should expand the effectiveness of this reovirus system.

## Methods

### Virus and cell lines

Reovirus ST3 strain Dearing and reovirus ST2 strain Jones were used. Both were grown in L929 mouse fibroblasts in MEM or RPMI supplemented with 5% FBS. The recombinant viruses containing the CAT gene (ST3.S2.CAT) were grown in L929 cells transformed with pHβ APr-1-neo [15] that contained ST3 S2 cDNA under the control of the human β-~actin promoter. The transformed cells, L-ST3.S2 cells, expressed protein σ2 at levels that were sufficient to rescue tsC 447 [16-18], a ST3 virus mutant with a ts mutation in the S2 genome segment, and support growth of recombinant CAT-containing reoviruses [10,12].

### Engineering of reovirus s2 cDNA

As previously demonstrated, we can incorporate an engineered reovirus s2 ssRNA into the reovirus genome as a stable dsRNA genome segment [10,12]. In this work, we deleted an internal 848 nucleotides from the wild type s2 sequence and replaced this with the CAT gene coding sequence (752 bp). This allowed us to distinguish between the wt s2 RNA and our engineered s2 RNA, both by sequence analysis and functional CAT activity. We have used this plasmid template to map the 5' sequences of the s2 ssRNA required to direct this ssRNA to be incorporated into a reovirus (6).

This template can be transcribed by T7 RNA polymerase to yield an RNA transcript that possesses 5' and 3' terminal sequences as authentic S2 RNA. The 5'-terminal S2 sequence ending at nucleotide 198 was fused, in frame, to the CAT gene coding sequence [12]. The 3' terminus of the CAT sequence was fused to the 3' S2 sequence, beginning at bp 1047 (of the wild type s2 RNA); and the 3' terminus of the S2 sequence, including the untranslated sequence, was fused to the Hepatitis Delta Virus (HDV) ribozyme sequence. Transcription by T7 RNA polymerase was terminated with the T7 terminator sequence located 3' of this construct. Transcription of this construction yielded an RNA that contained the 198 5' nucleotides of s2 RNA fused in frame to the CAT mRNA sequence followed by the 284 3' terminal nucleotides of s2 RNA. This was achieved by inserting the HDV ribozyme sequence in such a way that when the ribozyme underwent auto-cleavage, it left a terminal C at the 3' terminus. As a result, the 3' terminal sequence of the transcript was the authentic reovirus RNA 3' terminal sequence -UCAU**C**. Recloning and subsequent sequencing and cleavage analysis confirmed the authenticity of the 5' and 3' terminal sequences [12].

The pS2CAT198 construct was transcribed in vitro using T7 RNA polymerase and the transcript was capped using m^7 ^GpppG (Promega) to yield s2-CAT mRNA. It was translated in vitro using a rabbit reticulocyte lysate system (Promega) and the lysate was found to contain CAT activity (CAT-ELISA, Boehringer Mannheim Corporation).

### Mutagenesis of 3' s2 sequences down stream of the CAT gene in construct pS2CAT198 and pS2CAT96

Sequential deletion and mutagenesis of the 3' 284 nucleotides was carried out using GeneEditor™ by Promega (#Q9280). This system uses antibiotic selection to obtain a high frequency of mutants. Selection Oligonucleotides provided with the GeneEditor™ System encode mutations that alter the ampicillin resistance gene, creating a new additional resistance to the GeneEditor™ Antibiotic Selection Mix. As directed by the manufacturer, we annealed the selection oligonucleotide to our pS2CAT198 double-stranded DNA template at the same time as a mutagenic oligonucleotide. Subsequent synthesis and ligation of the mutant strand links the two oligonucleotides. The mutagenic oligonucleotides we selected were all 50 or more nucleotides in length, 25 nucleotides matching the 3' S2 and/or CAT nucleotide sequence depending upon the location of the sequence we wished to retain, and 25 nucleotides matching the HDV-ribozyme nucleotide sequence. Using 25 perfectly matched nucleotides on each side of the mismatched sequence we wished to loop-out and remove, we were able to remove nucleotides from the original pS2CAT198 sequence. For example, the sequence of the mutagenic oligonucleotide used to delete 50 nucleotides from pS2CAT96 to yield pS2CAT3b (Table 1) was; ^5'^GGCAGAAATTCGGATCCAAGATCTCCTCGAAACGTGGGCGAGAGAAGACG^3^. To replace the nucleotides in the plasmids generated from pS2CAT3m to yield templates such as pS2CATsub1 (Table 2) the mutagenic oligonucleotide was 60 nucleotides in length. For pS2CATsub1 the oligonucleotide contained 25 nucleotides matching pS2CATm sequences flanking 10 nucleotides that would be substituted for the wildtype sequences as summarized in Table 2 and shown in Figure 2.

### Removal of wt s2 RNA

Wild type s2 RNA was removed from the mixture of ten ssRNA species as previously described [19]. The DNA oligonucleotide selected for this purpose was complementary to nucleotides 937–949; and 10 pmoles were added to 2 pmoles of s2 RNA. After hybridization, the mixture was treated with RNAse H for 20 min. The RNA was extracted three times with phenol/chloroform and precipitated with sodium acetate. Degradation of the s2 RNA was confirmed by gel electrophoresis of both the RNA and its translation products. The set of nine ssRNAs was supplemented with the indicated s2-CAT RNAs and the resulting mixture was lipofected into L-ST3.S2 cells that were then infected with ST2 virus [11].

### The reovirus marker rescue system

The system was used as described [10-12], but modified to use L-ST3.S2 cells that express functional σ2 protein in place of L929 cells. ST3 capped and methylated mRNA (always referred to as ssRNA) was transcribed by cores [20]. After transcription, the cores were pelleted at 10,000 g; the supernatant, which contained the ssRNA was then made 0.5% with respect to sodium dodecyl sulfate (SDS) and extracted three times with phenol/chloroform. The RNA was precipitated with Polyethylene glycol (PEG), reextracted three times with penol/chloroform, and precipitated with 2.5 M ammonium acetate and ethanol. ssRNA prepared in this manner contained no residual infectious virus. For all lipofections, we used 10 μl of Rabbit Reticulocyte Lysate (Promega #L4960) primed with 0.3–0.5 μg of ST3 ssRNA (obtained from in vitro transcription from reovirus cores) and 0.1 μg of the indicated s2 ssRNA (obtained from in vitro transcription using T7 RNA polymerase and the indicated cDNA template, Promega- RiboMAX™-T7) in 1 μl H_2_O and 12 units of RNasin^® ^Plus RNase Inhibitor (Promega) in 1 μl H_2_O. Translation was allowed to proceed for 1 hour at 30 C. After translation, an additional 0.3–0.5 μg of ST3 ssRNA in 1 μl H_2_O was added and the mixture was immediately added to 0.5 ml of MEM containing penicillin and streptomycin and 50 μl of Lipofectin^® ^(Invitrogen Corporation). This mixture was immediately added to PBS-washed monolayers of 10^6 ^mouse L929 fibroblasts in 6-well multiplates. After 6 hr, this mixture was replaced with 0.25 ml of MEM containing 4 × 10^7 ^PFU of ST2 reovirus, and 1 hr later 1.75 ml of MEM containing 5% fetal bovine serum was added. After 24 hr, the cells were harvested, washed twice in MEM, and sonicated in 2 ml of MEM, and virus in the sonicates was titrated in mouse L929 fibroblast monolayers. To avoid detection of the ST2 helper virus, plaques were counted on day 5, when plaques formed by ST2 virus were not yet detectable.

### Virus titration/determination of CAT activity

Monolayers of lipofected and infected L-ST3.S2 cells were incubated at 37° for five days. Neutral red was added 24 hours before counting plaques [10,12]. CAT activity in cell lysates was assayed using CAT ELISA (Boehringer Mannheim Corporation). We measure the CAT activity of our engineered viruses as a method to screen large numbers of recombinant viruses when we encounter ssRNAs that are inefficiently incorporated in progeny viruses. Although useful this activity is not used to confirm that we have generated a recombinant virus. The CAT activity is low as it is expressed as a σ2-CAT fusion protein. The S2 dsRNA of all recombinant viruses is sequenced to confirm the presence of the S20CAT genome segment and it exact nucleotide sequence.

### Detection of reovirus s2 ssRNA in vivo

Twelve hours following lipofection of L929 or L-ST3.S2 cells with the indicated ssRNAs, protein translation mixture, and infection with reovirus serotype 2 helper virus, total RNA was extracted from the cell monolayers using Eppendorfs' Perfect RNA™ Eukaryotic Mini kit and protocols. The ssRNA was electrophoresed in a formaldehyde denaturing gel using Ambion, Inc's NorthernMax ^® ^kit. Following the manufacturer's protocol, the ssRNA was transferred to a BrightStar ^®^-Plus Positively Charged Nylon Membrane and UV-cross linked. Hybridization/detection was carried out at 40 C according to manufacturer's directions using UltrahybTM buffer and ^32^P-labelled oligonucleotides. For detecting the ST3 s2 ssRNA, the oligonucleotide (S2.5) ^5'^CAAACCACCAGACGTTTTACACGGTTAATTGCTGCTTGATA^3' ^complementary to nucleotides 55 to 95 near the 5' end of the s2 RNA was used. For detecting the s2/CAT ssRNA, the oligonucleotide (CAT.1) ^5'^TTTACGATGCCATTGGGATATATCGGTGGTATATCC^3' ^complementary to CAT gene was used. The oligonucleotide S2.5 was selected because it is 19.5% mismatched with the ST2 s2 sequence and, when used as a ssRNA probe, does not detect the ST2 s2 ssRNA or the S2 dsRNA genome segment. The membrane was used to expose x-ray film.

### Detection of reovirus s2 and CAT/s2 dsRNA in vivo

Twelve hours following lipofection of L929 or L-ST3.S2 cells with the indicated ssRNAs, protein translation mixture, and infection with reovirus serotype 2 helper virus, total cell monolayers were harvested. The dsRNA was electrophoresed in 7.5% SDS-PAGE gels for 2650 volt/hours as described (16). Following the protocol used for the ssRNA gels, the dsRNA was transferred to a BrightStar^®^-Plus Positively Charged Nylon Membrane and UV-cross linked. Hybridization/detection was carried out at 40 C according to manufacturer's directions, using UltrahybTM buffer and ^32^P-labelled oligonucleotides. For detecting the ST3 s2 dsRNA, the oligonucleotide S2.5 was used as described above. For detecting the s2/CAT ssRNA, the oligonucleotide CAT.1 complementary to CAT gene was used. The membrane was used to expose x-ray film.

### Recombinant virus purification

Forty-eight hours following lipofection of L929 or L-ST3.S2 cells with the indicated ssRNAs, protein translation mixture, and infection with reovirus serotype 2 helper virus, total cell monolayers were harvested. Serial 10-fold dilutions were prepared from these lysates and monolayers of L929 or L-ST3.S2 cells were infected. After incubation at 37° for five days, neutral red was added 24 hours before counting plaques [10,12]. Visualized plaques were selected using Pasteur pipettes and placed in 1 ml MEM w/o serum. Serial 10-fold dilutions were prepared from these individual virus isolates and monolayers of L929 or L-ST3.S2 cells were infected. After incubation at 37° for five days, neutral red was added 24 hours before counting plaques [10,12]. Visualized plaques were selected using Pasteur pipettes and placed in 1 ml MEM w/o serum. This plaque-purification process was repeated a third time. The dsRNA genomes of each three-time plaque-purified isolate were then analyzed by SDS-PAGE. In summary, individual wells of 96 well plates of L929 or L-ST3.S2 cells were infected with 50 μl of each isolate. After incubation at 37 C for 24 hours, 1 μCi ^32^P orthophosphate was added to each well. After an additional 24 hours, 50 μl 2X Laemmli sample buffer was added to each well and the plates stored at -20 C. The plates were heated to 65 C for 10 minutes and 10 μl of each well loaded onto a 7.5% SDS-PAGE gel and electrophoresis carried out for 2650 volt/hours. The gels were immediately dried and used to expose X-ray film. For recombinant viruses that should have the CAT gene expressed in frame, CAT activity was checked as an additional screen, and all recombinant reoviruses were identified by sequencing of the engineered S2 dsRNA to confirm that the engineered ssRNA made in vitro had been incorporated as constructed.

### Sequencing of cDNA templates and recombinant viruses

All cDNA templates were sequenced to confirm the presence of the desired mutations. The T7-generated ssRNAs were sequenced using two methods: the 5' 200 nucleotides sequenced using reverse transcriptase (RT) and a complementary primer, the 3' ends first poly-A tailed using yeast poly-A polymerase, then sequenced using RT and an oligo-T primer, as described [10,12,21]. Following purification, all recombinant reoviruses were propagated and the S2 dsRNA genome segments sequenced directly using reverse transcriptase as described [21].

## Authors' contributions

MRR and JR constructed the cDNA templates, generated and sequenced the engineered viruses, preformed the northerns, CAT assays and SDS-PAGE gel analysis. MRR is the principal investigator and wrote the manuscript.
